# Comparative QTL mapping for male sterility of cultivated strawberry (*Fragaria* × *ananassa* Duch.) using different reference genome sequences

**DOI:** 10.1270/jsbbs.20151e

**Published:** 2022-04

**Authors:** Takuya Wada, Hiyori Monden, Sachiko Isobe, Kenta Shirasawa, Takayuki Sueyoshi, Chiharu Hirata, Miyuki Mori, Shiro Nagamatsu, Yoshiki Tanaka

**Affiliations:** 1 Department of Agro-environment, Fukuoka Agricultural and Forestry Research Center, 587 Yoshiki, Chikushino, Fukuoka 818-8549, Japan; 2 Department of Frontier Research, Kazusa DNA Research Institute, 2-6-7 Kazusa-kamatari, Kisarazu, Chiba 292-0818, Japan

Breeding Science 71: 456–466 (2021)

In the above article, accession numbers of raw reads, which were used in this study and registered in DDBJ Sequenced Read Archive, were not mentioned. Therefore, we herewith would like to publish the correct Materials and Methods section indicating DRA number of the reads.

## False

### Cleaning raw reads, mapping, and variant calls

The fastq files that contained the obtained raw reads were trimmed and cleaned with Trimmomatic software v. 0.39 (Bolger *et al.* 2014). Sequences were mapped to the reference sequences in BWA software (Li and Durbin 2009) separately using ‘Camarosa’ genome assembly v. 1.0.a1 (Edger *et al.* 2019b) and ‘Reikou’ genome assembly r2.3 (https://www.biorxiv.org/content/10.1101/2021.04.23.441065v1). The constructed sequence alignment map (sam) files were converted and sorted to produce binary alignment map (bam) files, then the duplicated reads generated as PCR duplication artifacts were excluded from the sorted bam files by the Markduplicates function of Picard tools (http://broadinstitute.github.io/picard). Variant calls were performed using the bam files for F_bulk and S_bulk in samtools software (https://github.com/samtools) and bcftools software (https://github.com/samtools/bcftools) to generate variant call format (vcf) files, then the generated vcf files were converted to tidy data to obtain comma-separated value (csv) files in the vcfR package for R software (Knaus and Grünwald 2017). The alternate allele frequency (Alt_AF) for each single-nucleotide polymorphism (SNP) position in F_bulk and S_bulk was calculated with the dplyr package for R software (Wickham *et al.* 2019), then we calculated a moving average of Alt_AF with the simpleSmoothTs command of the latticeExtra package (Sarkar and Andrews 2016) using Alt_AF values of the 1,000 front and 1,000 back SNPs. The regions where the difference in Alt_AF values between the two bulk populations was greater than the threshold value (*P* < 0.01) were designated as candidate genomic regions for the relevant QTL.

## True

### Cleaning raw reads, mapping, and variant calls

The fastq files (DDBJ Sequence Read Archive no. DRA010813) that contained the obtained raw reads were trimmed and cleaned with Trimmomatic software v. 0.39 (Bolger *et al.* 2014). Sequences were mapped to the reference sequences in BWA software (Li and Durbin 2009) separately using ‘Camarosa’ genome assembly v. 1.0.a1 (Edger *et al.* 2019b) and ‘Reikou’ genome assembly r2.3 (https://www.biorxiv.org/content/10.1101/2021.04.23.441065v1). The constructed sequence alignment map (sam) files were converted and sorted to produce binary alignment map (bam) files, then the duplicated reads generated as PCR duplication artifacts were excluded from the sorted bam files by the Markduplicates function of Picard tools (http://broadinstitute.github.io/picard). Variant calls were performed using the bam files for F_bulk and S_bulk in samtools software (https://github.com/samtools) and bcftools software (https://github.com/samtools/bcftools) to generate variant call format (vcf) files, then the generated vcf files were converted to tidy data to obtain comma-separated value (csv) files in the vcfR package for R software (Knaus and Grünwald 2017). The alternate allele frequency (Alt_AF) for each single-nucleotide polymorphism (SNP) position in F_bulk and S_bulk was calculated with the dplyr package for R software (Wickham *et al.* 2019), then we calculated a moving average of Alt_AF with the simpleSmoothTs command of the latticeExtra package (Sarkar and Andrews 2016) using Alt_AF values of the 1,000 front and 1,000 back SNPs. The regions where the difference in Alt_AF values between the two bulk populations was greater than the threshold value (*P* < 0.01) were designated as candidate genomic regions for the relevant QTL.

